# Determination of Enantiomeric Excess in Confined Aprotic Solvent

**DOI:** 10.1021/acselectrochem.4c00219

**Published:** 2025-02-26

**Authors:** Emer B. Farrell, Fionn McNeill, Dominik Duleba, Adria Martínez-Aviño, Patrick J. Guiry, Robert P. Johnson

**Affiliations:** School of Chemistry, 8797 University College Dublin, Belfield, Dublin D04 N2E5, Ireland

**Keywords:** Enantiomeric Excess, Enantiopurity, Ion-Current Rectification, Nanopore, Aprotic Solvent

## Abstract

The validation of the stereochemical purity of synthesized compounds is a requisite for the fine-chemical industry, particularly in the production of enantiopure drug compounds. However, most methodologies employed in the determination of enantiopurity require carefully chosen chiral GC or HPLC columns, increasing associated cost, analysis time, and complexity. Herein, we present a nanopore-based technology for the determination of enantiopurity, exploiting changes in ion-current rectification of quartz nanopipettes containing an aprotic organic electrolyte. Changes in solvent ordering at the quartz surface upon enantiomerically preferential adsorption give rise to distinguishable current-voltage responses. The applicability of our simple and cost-effective platform is demonstrated through the determination of the enantiomeric excess of commercially available (*R*)- and (*S*)-enantiomers of 4-methoxy-α-methylbenzylamine and duloxetine hydrochloride, as well as the product of a decarboxylative asymmetric allylic alkylation. Ion-current rectification (ICR)-based enantiomeric excess determination is completed within minutes, using negligible sample volumes and with simple low-cost electrical instrumentation.

## Graphical abstract

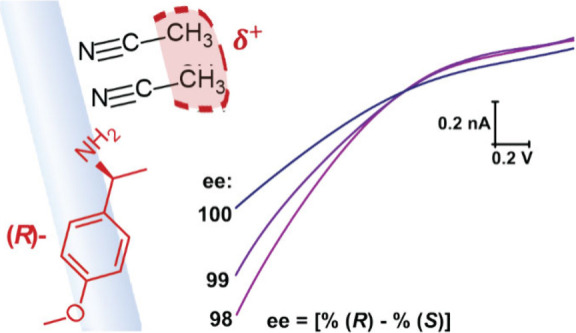

## Introduction

The chemical industry has moved away from the production of racemic drug compounds, and toward enantiopure synthesis, due to the (sometimes drastically) differing bioactivity of (*R*)- and (*S*)-enantiomers.^1^ A well-known, early example of this is thalidomide, a drug used to treat morning sickness in pregnant women in the late 1950s,^2^ which has the desired biological effect in the (*R*)-form, but causes teratogenicity (birth defects) in the (*S*)-form.^3^ In many cases, one enantiomeric form of a drug has much higher activity than the other. For example, (*S*)-Citalopram, a selective serotonin reuptake inhibitor (SSRI) used to treat depression, is 100 times more potent than (*R*)-citalopram.^4^ Accordingly, the measurement of the enantiopurity of drug compounds is essential for the quality control of pharmaceuticals.^5^

Enantiomers exhibit unique optical properties, which can be utilized for their direct detection based on either their rotation of plane-polarized light (optical rotation/polarimetry), or their absorption of circularly polarized light (circular dichroism).^6,7^ Polarimetry, however, is seldom used in practice, and is significantly disadvantaged by low sensitivity, intolerance to impurities and issues with reproducibility.^6,8^ A study by Joyce *et al*.^9^ described how discrepancies in the optical rotation values of a natural product may arise due to the presence of minor amounts of impurities (with stronger optical rotation). The authors regarded the sole use of optical rotation responsible for incorrect conclusions regarding the absolute configuration of the natural product.^9^ In some cases, including where compounds have low specific rotation values, optical rotation cannot be used to determine enantiopurity, and is associated with high uncertainty.^10,11^ Less disadvantages are reported for circular dichroism, besides the pre-requisite for strongly absorbing chromophores, which many chiral organic compounds do not possess.^12,13^ For this reason, circular dichroism is often induced by derivatization with strongly absorbing host molecules.^12,14–16^ Circular dichroism and polarimetry are rarely used alone to determine enantiopurity, and are more commonly incorporated as detectors in high-performance liquid chromatography (HPLC) instruments.^8,13,17,18^

Enantiopurity in practice is typically determined using a number of analytical techniques, including nuclear magnetic resonance (NMR) spectroscopy,^19,20^ capillary electrophoresis (CE),^21,22^ and chiral high-performance liquid chromatography (HPLC) and gas chromatography (GC).^23,24^ However, these techniques are limited by their complexity and their requirement for expensive instrumentation and trained personnel.^25^ Additionally, they require the presence of a carefully-crafted chiral environment to discriminate enantiomers. For example, chiral HPLC relies on chiral acceptor molecules incorporated into the stationary phase,^26^ and NMR spectroscopy requires chiral derivatizing agents to convert enantiomers into distinguishable diastereomers.^27^

Electrochemical sensors have also been explored for chiral discrimination, due to their simplicity, low-cost, and potential sensitivity.^5^ These sensing devices comprise an electrode surface modified with a chiral reagent to produce an enantioselective interface, giving an enantio-dependent change to the current magnitude.^25^ An interesting example of this technique was reported by Assavapanumat *et al*.^28^ who used a chiral imprinted mesoporous Ni electrode to discriminate between (*R*)- and (*S*)-phenylethanol based on current density using differential pulse voltammetry. The same researchers also used this technique with an imprinted mesoporous Pt electrode for the enantioselective recognition of l- and d-tryptophan.^29^ Furthermore, Arnaboldi and coworkers have developed inherently chiral oligomers by electropolymerization of an enantiopure benzothiophene monomer,^30^ implementing this material in a range of bipolar electrochemical systems for the wireless chiral discrimination of amino acids.^5,31,32^ More traditional approaches include the functionalization of electrode surfaces with chiral probe molecules, such as β-cyclodextrin and chiral metal organic frameworks (MOFs), which are described in a recent review by Salinas *et al*.^25^ All of these examples require either enantiopure materials or probe molecules. This requirement is a significant limitation, and increases the cost, complexity and time-consumption of sensor fabrication.

In recent years, our group have explored the use of easily fabricated quartz nanopipettes as electrochemical sensors, based on changes to ion transport through the nanopore in the presence of analyte. Under an internal applied potential, given the nanopore exhibits some aspect of asymmetry and possesses a surface charge, non-ohmic current-voltage (*I–V*) traces are observed. This is known as ion-current rectification (ICR), and arises due to electrical double layer (EDL) overlap, and resulting perm-selectivity, at the nanopore tip.^33,34^ By binding probe molecules to the nanopore wall, surface charge can be modulated in the presence of an analyte, for ICR-based sensing applications.^35^ Han *et al*.^36^ reported the use of β -cyclodextrin-modified nanopores for the chiral discrimination of l- and d-histidine based on ICR. The same group have also described the use of l- and d-cysteine-modified nanopores to modulate bovine serum albumen (BSA) translocation, and explore the role of chirality in biological protein translocation events.^37^ To date, the majority of work on ICR is in aqueous electrolyte, and few reports describe nanopore behavior in an aprotic organic solvent.^38–42^

In our previous work, we reported the unusual ion current rectifying properties of bare quartz nanopipettes in aprotic solvent at low supporting electrolyte concentrations, showing that under specific conditions, accumulation of the aprotic solvent and the subsequent formation of double-junction diodes within the nanopipette gives rise to unexpectedly high rectification ratios.^38^ This followed earlier reports by Plett *et al*.^40^ and Yin *et al*.^39^ who proposed that the surface charge of nanopores filled with aprotic solvent arises due to the dipole ordering of the solvent molecules along the neutral nanopore wall. In a recent study, Silva *et al*.^41^ also demonstrated chirality-controlled ion transport through nanopores, due to the different effective surface charge imparted by racemic vs. chiral propylene carbonate, as well as emergence of chiral electrokinetic phenomena.^42^ These phenomena were shown to occur as a direct consequence of solvent structure at the nanopore wall.^41^

Herein, we present a nano-electro-chemical system for the determination of enantiomeric excess (ee) based on ion-current rectification (ICR). While in the previous works noted above different chiral solvents were used to modulate ICR, the work we present here uses an achiral solvent media into which a chiral analyte of interest is added, forming the basis of an enantioselective sensing device. The observed differences in ICR response for (*R*)- and (*S*)-enantiomer analyte are explained in terms of differing aprotic solvent ordering upon adsorption of the enantiomers under study to the pore walls, which in turn drives changes to the internal surface charge of quartz nanopipettes. We demonstrate that our technology can be used to determine the enantiomeric excess of the product of a Pd-catalyzed decarboxylative asymmetric allylic alkylation within minutes, paving the way for its potential use for quality control in pharmaceutical production.

## Experimental Section

### Materials

Quartz capillaries (0.7 mm I.D., 1 mm O.D., Sutter Instruments) were used for the fabrication of the quartz nanopipettes. The electrolyte employed in ICR measurements was tetraethylammonium tetrafluoroborate (99%, Alfa Aesar) or sodium tetrafluoroborate (97%, Thermo Scientific) dissolved in acetonitrile (99.9%, Fisher Scientific). Acetonitrile for nanopipette measurements was in all instances used “as-is”, without drying or further purification. Nanopipette radii were measured using potassium chloride (99%, Acros Organics) dissolved in Milli-Q water with Ag/AgCl wires (prepared using Ag wires (99.9%,Merck)) as working and reference electrodes. Pt wires (99.9%, Merck) were used as electrodes in organic electrolyte systems. (*R*)-4-Methoxy-α-methylbenzylamine (99%, Merck), (*S*)-4-methoxy-α-methylbenzylamine (98%, Merck), (*R*)-duloxetine hydrochloride (98%, ChemCruz) and (*S*)-duloxetine hydrochloride (98%, TCI) were used as purchased. The Pd-catalyzed reactions were carried out with rigorous exclusion of air and moisture under an inert atmosphere of nitrogen in flame-dried glassware with magnetic stirring un-less otherwise stated. N_2_-flushed plastic syringes were used to transfer air and moisture sensitive reagents. Oxygen free nitrogen was obtained from BOC gases. (*R*,*R*)-ANDE*N*-phenyl Trost ligand was purchased from BLDpharm and used as received. Anhydrous 1,4-dioxane was obtained from Thermo Fischer Scientific and used as received. Tris­(dibenzylideneacetone)­palladium(0) chloroform adduct was prepared via the method of Ananikov.^43^ In vacuo refers to the evaporation of solvent under reduced pressure on a rotary evaporator. Flash column chromatography was performed using 40-63 μm, 230-400 mesh silica gel.

### Nanopipette Fabrication and Characterization

Nanopipette fabrication was carried out using a Sutter P-2000 micropipette puller with 5 tunable parameters heat (H), filament (F), velocity (V), delay (D) and pull (P). The following program was employed to fabricate 50 nm nanopipettes (program 70, Line 1: H700, F4, V20, D170, P0, Line 2: H680, F4, V50, D170, P200) from 0.7 mm quartz capillaries. This program fabricates pores of 49 ± 2 nm and is a pore geometry for which we have previously characterized in detail in the ion-rectification behaviour in aprotic solvent systems. The size of the nanopipettes was characterized by conductivity measurement and scanning electron microscopy (Figure S1), as described in detail in the Supporting Information.

### ICR Measurements

Nanopipettes were backfilled with 0.5 mM tetraethylammonium tetrafluoroborate (TEATFB) in acetonitrile (MeCN) unless stated otherwise. These supporting electrolyte and solvent conditions were chosen because we have previously demonstrated that the ICR response of nanopipettes in this media is stable and reproducible.^38^ A Pt wire working electrode was inserted into the nanopipettes, which were placed in a bulk electrolyte bath of the same concentration containing a Pt wire reference electrode. For enantiomer detection, the current voltage (*I*–*V)* response was measured in a 0.5 mM TEATFB bulk electrolyte bath containing equimolar chiral solute. *I–V* traces were measured using a Biologic SP-200 potentiostat with an ultra-low current probe. The applied potential was swept from -1 to 1 V with respect to the reference electrode, at a scan rate of 0.1 V s^–1^. Measurements were performed with a filter bandwidth of 5 Hz to remove noise, and absolute current values at +1 V and -1 V were extracted from polynomial fits. Calculation of rectification ratio, (RR) accounting for *y*-offset values (which we have observed in organic ICR measurements using Pt wire electrodes) is shown in eq 1, where |*I* ^‑^| is the absolute current at −1 V, and |*I* ^+^| is the absolute current at +1 V. *I*_y–off_ represents the actual current at 0 V (y-offset value). Accordingly, a negative *y*-offset (-*I*_y–off_) results in an increased |*I* ^+^| and a decrease*d* |*I* ^–^|, while a positive y-offset (+*I*_y–off_) results in a decreased |*I* ^+^| and an increased |*I* *^–^* |.

1 $$\mathrm{RR}=\frac{\left|I^{-}\right|+I_{y\text{−}\mathrm{off}}}{\left|I^{+}\right|-I_{y\text{−}\mathrm{off}}}$$

Rectification ratio values in this work are presented as mean ± standard error of the mean from a minimum of six measurements. Each measurement represents a unique, single use nanopipette sensor device.

## Results and Discussion

### Enantio-Discrimination with Conical Quartz Nanopipettes

In acetonitrile (MeCN), at a tetraethylammonium tetrafluoroborate (TEATFB) electrolyte concentration of 0.5 mM, quartz nanopipettes display negative ICR. This is described in our previous work, and arises due to the accumulation or depletion of MeCN solvent molecules along the inner surface of the nanopore in regions where ions accumulate or deplete.^38^ By addition of an equimolar amount of an organic compound, we hypothesized that this accumulation or depletion behavior could be altered through disruption of MeCN solvent ordering, changing the rectifying behavior of the nanopore. Figure 1a shows the current-voltage (*I–V)* response of bare quartz nanopipettes in a bulk electrolyte bath, relative to the response obtained when the electrolyte additionally contains either 0.5 mM (*S*)- or (*R*)-4-methoxy-α-methylbenzylamine. Addition of a chiral compound drives notable changes in the rectification exhibited by the pore, i.e., the extent to which the *I–V* response deviates from linearity, a property which can be characterized by the rectification ratio (RR), i.e., the ratio of the current magnitude at equal but opposite voltages, calculated as described in the Experimental Section (eq 1).

**Figure 1:**
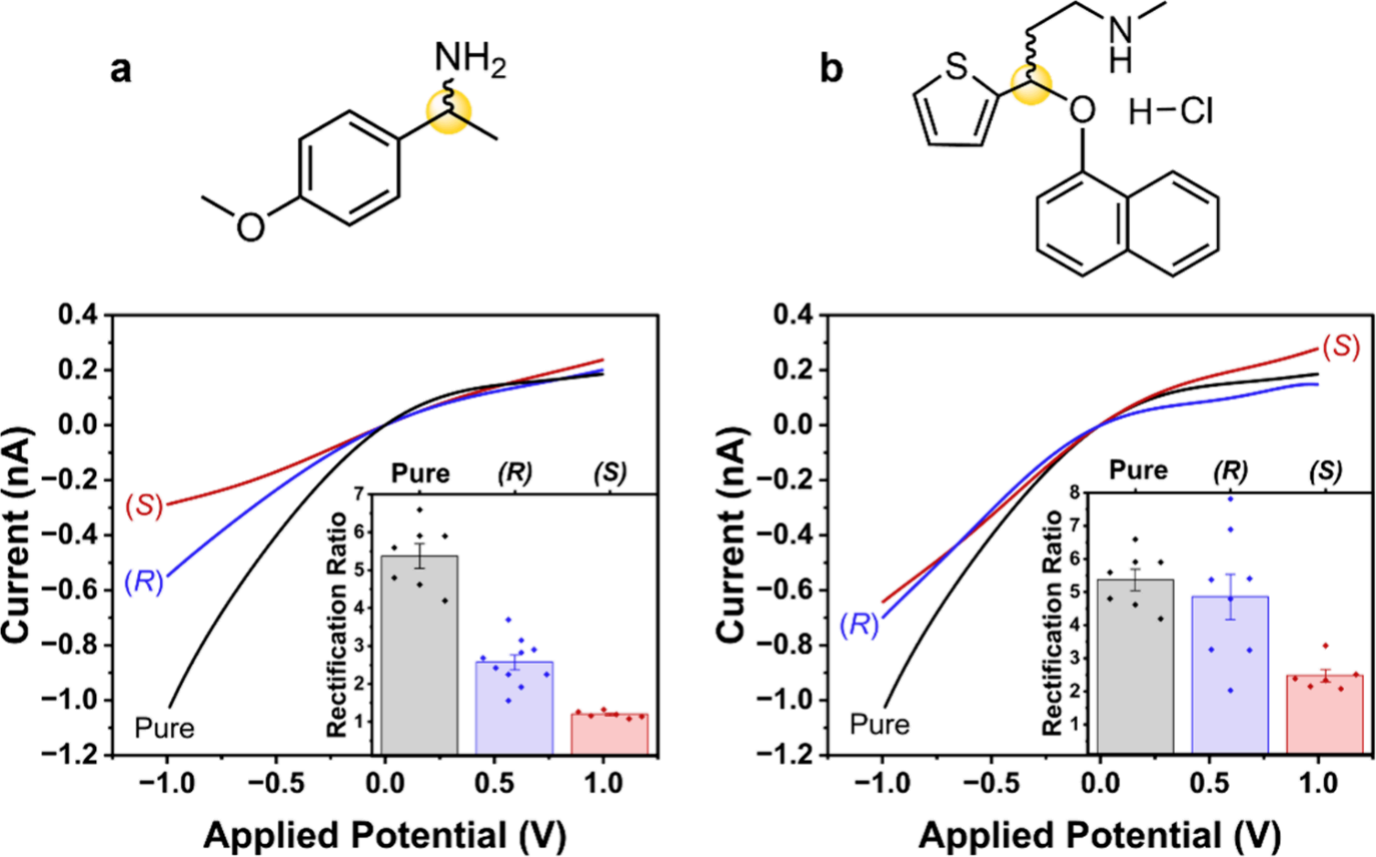
Chemical structure (with chiral center highlighted) and representative *I–V* responses (showing rectification ratio inset). Rectification ratio is measured in (black) pure electrolyte and electrolyte containing (blue) (*R*)- and (red) (*S*)-enantiomer for (a) 4-methoxy-α-methylbenzylamine and (b) duloxetine hydrochloride. *I–V* responses are measured in 0.5 mM TEATFB in MeCN, using bare quartz nanopipettes with radii of ∼50 nm. 0.5 mM enantiomer is added to the external bulk electrolyte bath for detection. Representative error bars indicate the standard error from a measurement of six or more unique nanopipettes, and replicates showing the variability in response across unique nanopipette devices are shown in Figure S2. The stability of the *I–V* response with respect to time is shown in Figure S3.

The rectification ratio of the measured nanopipettes, in the absence and presence of either 0.5 mM (*S*)- or (*R*)-4-methoxy-α-methylbenzylamine are shown inset in Figure 1a. A rectification ratio (RR) of 5.4 ± 0.3 is measured in bare quartz nanopipettes in TEATFB, while measurements with 4-methoxy-α-methylbenzylamine in the external bulk electrolyte bath show significant changes to ICR, which is dependent on the enantiomeric form of the compound. In the presence of the (*S*)-enantiomer ICR is significantly suppressed to 1.20 ± 0.03, while in the presence of the (*R*)-enantiomer a less significant change to 2.6 ± 0.2 is observed. The second compound investigated was duloxetine hydrochloride, a selective serotonin and norepinephrine reuptake inhibitor (SSNRI) used to treat depression, anxiety, and pain associated with diabetes.^44^ It is a drug marketed only in its (*S*)-form, which is twice as effective as the (*R*)-form.^45^ Figure 1b shows the enantiomeric discrimination of duloxetine hydrochloride using the procedure described above. In the presence of the (*S*)-enantiomer ICR is suppressed to 2.5 ± 0.2, while in the presence of the (*R*)-enantiomer a minimal change to 4.9 ± 0.6 is observed. Interestingly, duloxetine suppresses ICR to a much lesser degree than 4-methoxy-α-methylbenzylamine, indicating the importance of the chemical structure to MeCN solvent ordering. For both sets of enantiomers, and for all enantiomeric forms, a suppression of the ICR is observed relative to ICR of a nanopore containing pure electrolyte.

Following from the successful discrimination of (*R*)- and (*S*)-4-methoxy-α-methylbenzylamine and duloxetine hydrochloride, the enantiomers were mixed in varying ratios, defined based on enantiomeric excess (ee), and ICR analysis was carried out on each solution:

ee=%(R)‐enantiomer−%(S)‐enantiomer

For both species we found that a trend was observed only where the (*R*)-enantiomer was in excess. Due to the major suppressive effect of the (*S*)-enantiomer on ICR, we believe the presence of any minor amount of the lesser impactful (*R*)-enantiomer has a negligible effect. In contrast, where (*S*) is the minor enantiomer, significant changes to ICR are observed as a function of increasing excess of the (*R*)-enantiomer. Figure 2 shows the change in RR as a function of ee [% (*R*) – % (*S*)] for 4-methoxy-α-methylbenzylamine. At ee values above 50%, ICR becomes more negative, and the rectification ratio increases towards a maximum at 70%. After this maximum, an exponential decay is observed towards 100%.

**Figure 2:**
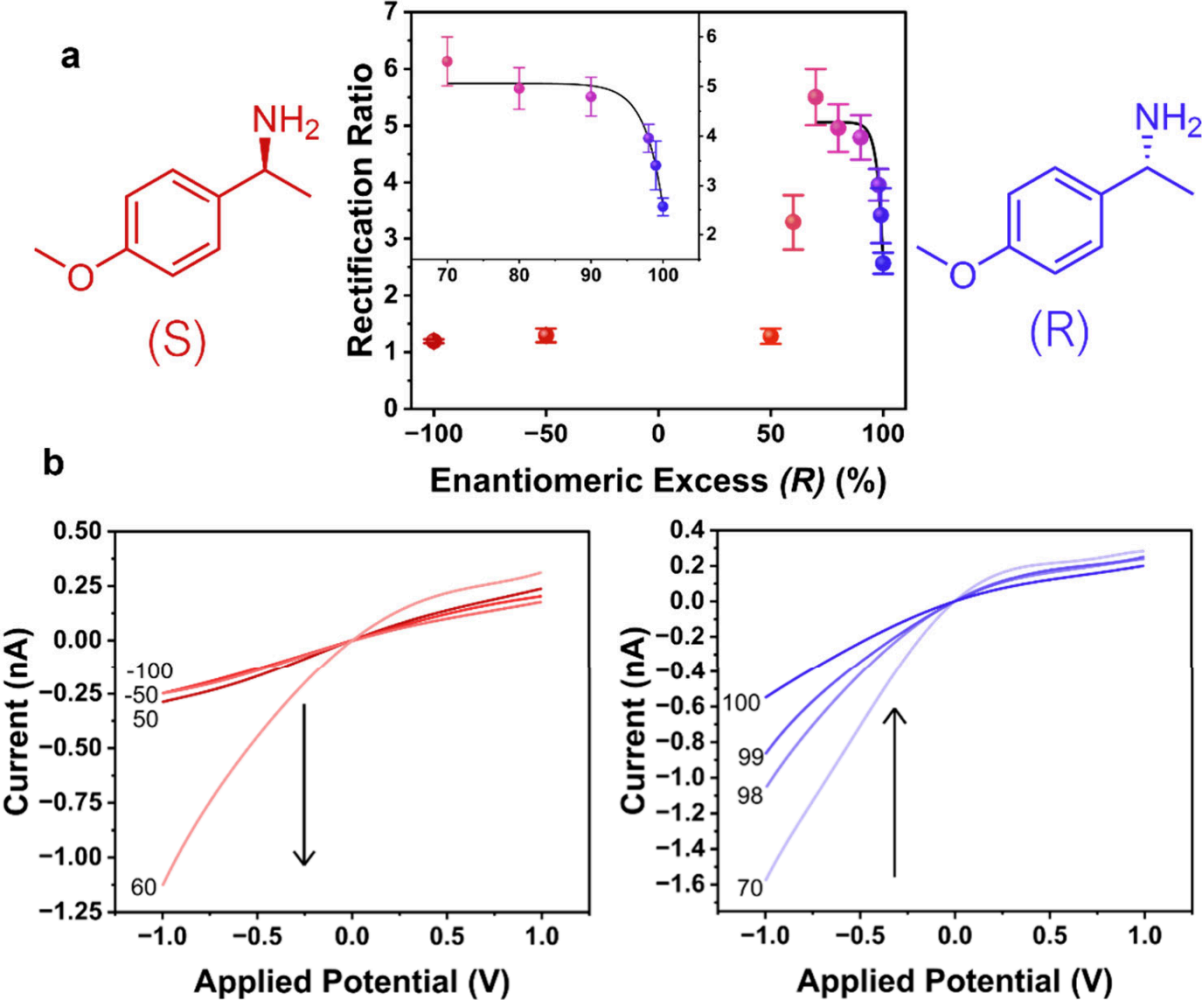
(a) Rectification ratio as a function of enantiomeric excess of (*R*)-4-methoxy-α-methylbenzylamine [% (*R*) – % (*S*)] and (b) representative *I–V* responses for various enantiomeric excess values. All *I–V* responses are measured in 0.5 mM TEATFB in MeCN, using bare quartz nanopipettes with radii of ∼50 nm. 0.5 mM enantiomer is added to the external bulk electrolyte bath for detection. Representative error bars indicate the standard error from a measurement of six or more unique nanopipettes. Samples of different enantiomeric excess are achieved by mixing stock solutions of commercially available (*R*)- and (*S*)-4-methoxy-α-methylbenzylamine in different ratios, with dilution to an overall analyte concentration of 0.5 mM.

This type of observation is not unusual in aprotic electrolyte systems, whose surface charge is nonlinear, and whose ICR is dependent on the accumulation/depletion of solvent molecules in regions of ion accumulation/depletion, as our group has previously established.^38^ Accordingly, changes in the surface charge pattern along the nanopore wall in the presence of an analyte can give rise to nonlinear changes in RR as a function of concentration. As the concentration of the (*S*)-enantiomer decreases, a maximum is reached, after which a more “classical” decay in signal is observed.

Figure 3 shows the same analysis carried out on duloxetine hydrochloride, where a similar trend is observed.

**Figure 3:**
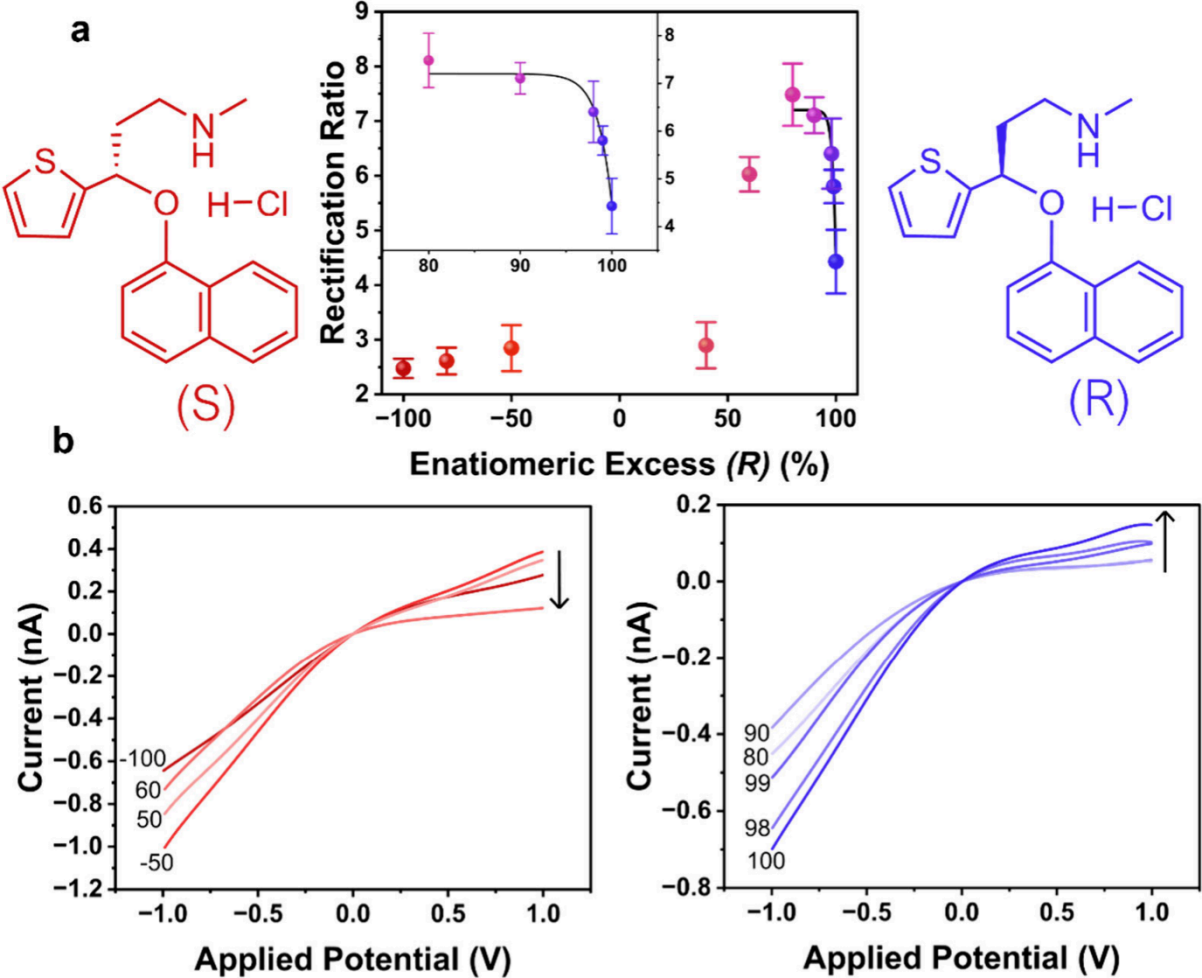
(a) Rectification ratio as a function of enantiomeric excess of (*R*)-duloxetine hydrochloride [% (*R*) – % (*S*)] and (b) representative *I–V* responses for various enantiomeric excess values. All *I–V* responses are measured in 0.5 mM TEATFB in MeCN, using bare quartz nanopipettes with radii of ∼50 nm. 0.5 mM enantiomer is added to the external bulk electrolyte bath for detection. Representative error bars indicate the standard error from a measurement of six or more unique nanopipettes. Samples of different enantiomeric excess are achieved by mixing stock solutions of commercially available (*R*)- and (*S*)-duloxetine hydrochloride in different ratios, with dilution to an overall analyte concentration of 0.5 mM.

### Mechanism of Enantio-Selectivity in Quartz Nanopipettes

The origin of the apparent enantiomeric selectivity of unmodified nanopipettes in aprotic solvent is not immediately obvious, because the pore surfaces themselves have not been modified with any rationally designed chiral selector. The electrolyte and solvent within the pore, in which the enantiomers are dissolved is also achiral. Our results therefore suggest that in aprotic solvent, the surfaces of the quartz nanopipette are themselves chiral selective, or, more precisely, contain chiral-selective adsorption or binding sites. In further experiments, we found that nanopipettes exposed to either enantiomer of 4-methoxy-α-methylbenzylamine, which were then subsequently rinsed and re-measured in pure electrolyte, retained their suppressed RR values (Figure S4). These data indicate that suppression of ICR in nanopipettes containing aprotic solvent arises from adsorption of the enantiomer analyte to the walls of the nanopipettes and is not generated by the presence of the enantiomer analyte in the solution phase. Further experiments also showed that the extent of RR suppression observed upon exposure to either the (*R*)- or (*S*)-enantiomer, as well as the ability to discriminate the enantiomer pair, were also dependent on the exposure concentration (Figure 4).

**Figure 4:**
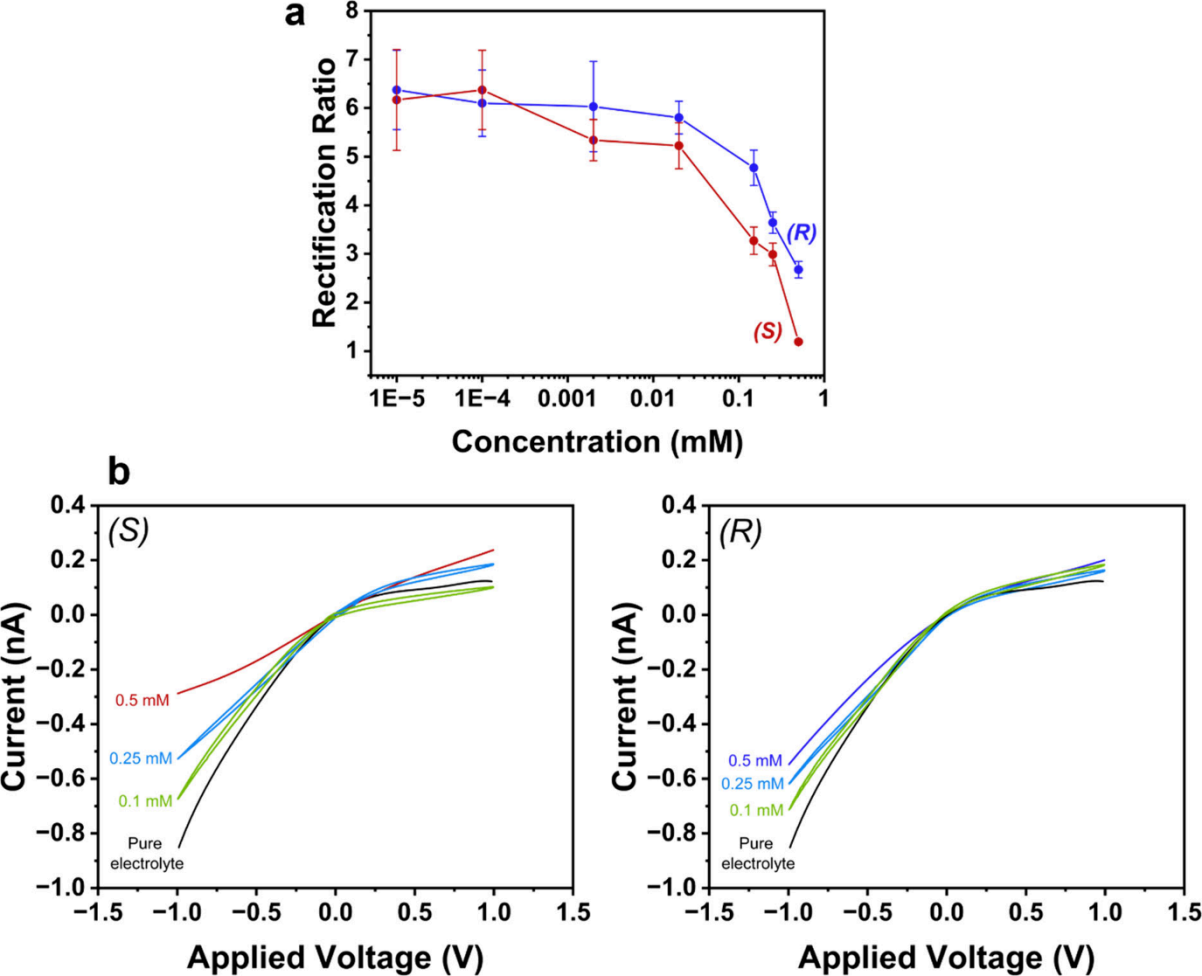
(a) Change in rectification ratio as a function of the concentration of (*S*)- and (*R*)-4-methoxy-α-methylbenzylamine and (b) representative *I–V* responses for the enantiomers at different concentrations. *I–V* responses are measured in 0.5 mM TEATFB in MeCN, using bare quartz nanopipettes with radii of ∼50 nm. 0.5 mM, 0.25 mM, or 0.1 mM enantiomer is added to the external bulk electrolyte bath for detection. Representative error bars indicate the standard error from a measurement of six or more unique nanopipettes.

Given that adsorption of both the (*R*)- and (*S*)-forms to the internal walls of a nanopipette in aprotic solvent is observed, and, that this adsorption manifests as an apparent suppression of the rectification, we speculate that the interior surfaces of the quartz nanopipette contains binding sites that are preferential for both (*R*)- and (*S*)-enantiomers, with the greater RR suppression observed for (*S*)- suggesting that the internal walls of the nanopipette contain a higher number of (*S*)- preferential binding sites. The origin of these chiral-selective binding sites at the surfaces of the quartz nanopipettes is unclear but may result from impurities introduced during the laser-pull fabrication process, or itself be an intrinsic feature of the nanopipette’s quartz surfaces. Quartz is a well-established chiral material,^46,47^ and quartz surfaces have been reported to promote enantiomeric selectivity,^48^ as well as preferential enantiomeric adsorption from non-aqueous solvents,^49–56^ therefore such a mechanism would fit well with existing literature. Furthermore, the confinement of the nanopipette environment (the pore here is ∼50 nm diameter) may promote adsorption of all molecules at the interface, and in future work, we intend to investigate the impact of pore size on the ability to discriminate enantiomers using our system.

Taking into account these earlier works, we tentatively propose the following sensing mechanism. For a bare nanopipette in MeCN, the surface silanol groups on the quartz nanopore walls remain protonated, with exposed, partially positive (δ^+^) H terminal groups. Interaction between nearby MeCN solvent molecules and the H terminal groups result in the ordering of MeCN molecules along the nanopore wall, such that their δ^+^ CH_3_ groups point outwards. This gives rise to an effective positive surface charge (Figure 5a), and the complex ICR behaviour previously reported by our group,^38^ and others.^39,40^ We previously hypothesized that the disruption of this solvent ordering in the presence of an analyte of interest in the solution phase could be exploited for probe-free nanopore sensing, and demonstrated this by monitoring the photoisomerization of spirooxazine to merocyanine in MeCN with bare quartz nanopipettes.^57^ In the current work, suppression of ICR in the presence of both (*S*)- or (*R*)-4-methoxy-α-methylbenzylamine arises from adsorption of the enantiomer to the nanopipette walls (Figure S4). The greater degree of suppression that occurs in the presence of (*S*)-enantiomers is suggestive of a larger number of preferential (*S*)-binding sites. It then follows that the presence of an (*S*)-enantiomer in the bulk electrolyte results in more significant changes to MeCN solvent ordering along the nanopore wall, as less of the “pure” quartz surface remains exposed (Figure 5b). Conversely, In the presence of an (*R*)-enantiomer, where we propose there are fewer preferential binding sites, the change in ICR is smaller as more of the “pure” quartz surface remains exposed (Figure 5c). It is important to note that the tentative mechanism we propose here is simplified. ICR in aprotic solvent is complex due to the formation of double-junction diodes,^38^ and this likely impacts the nanopore’s behavior in the presence of enantiomers, which may themselves adsorb to the nanopipette walls non-homogeneously across the internal pore walls, further magnifying any diode effect.

**Figure 5:**
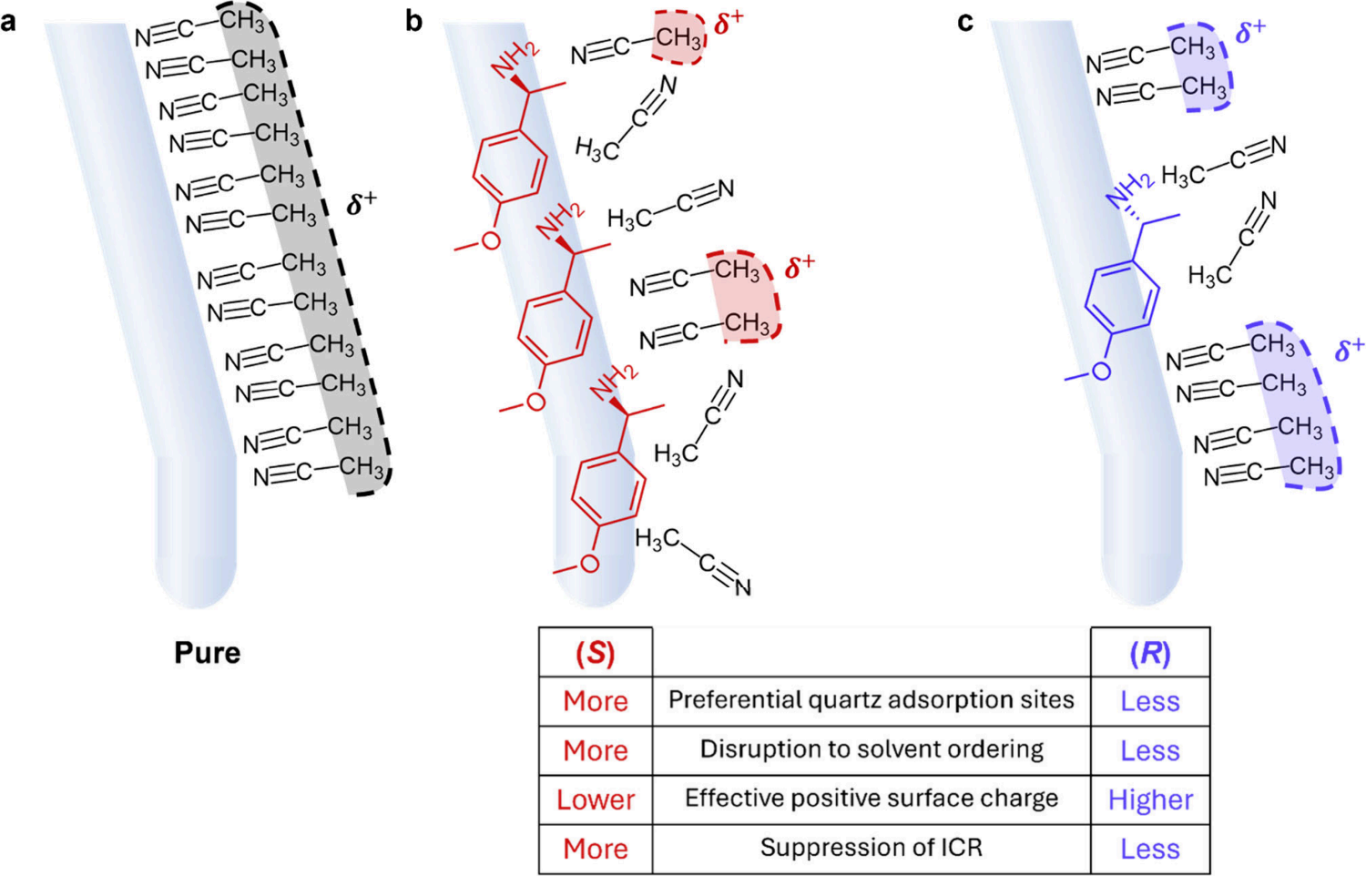
A simplified schematic showing (a) the effective positive surface charge of a quartz nanopipette in pure MeCN electrolyte that arises due to solvent ordering, where (b) a larger number of (*S*)-adsorption sites on the quartz surface may result in more extensive disruption of MeCN solvent ordering and, as a result, a lower effective positive surface charge and suppressed ICR. (c) A lower number of (*R*)-adsorption sites may result in less suppression of ICR, as the “pure” quartz surface is more exposed.

The tentative mechanism we are proposing, which relies on the presence of H terminal groups at the internal walls to drive solvent ordering, will be strongly affected by the presence of even trace water. Furthermore, established literature suggests that adsorption of enantiomeric amino acids onto quartz surfaces from aqueous solvent is minimal,^58,59^ with work by Bonner and co-workers half a century ago concluding that non-aqueous solvents with rigorous control of moisture are required for reproducible asymmetric adsorption onto quartz surfaces.^50,59^ In our work, we found that a relatively dry (∼< 50 ppm) solvent is required for the nanopipettes to exhibit chiral selectivity (Figure 6). The typical water content of the electrolyte used in the experiments we report here, as measured by Karl-Fisher titration, was found to be ∼30 ppm. Electrolytes prepared for the measurements presented here were done so with commercially purchased solvent used “as-is”, i.e., without pre-treatment or drying. Under these relatively dry solvent conditions, (*R*)- and (*S*)-enantiomers are clearly distinguishable by ICR. However, additional doping of the electrolyte with water does result in a loss of discrimination, with the rectification ratio of nanopores containing pure electrolyte, (*R*)- or (*S*)-enantiomers all converging to an RR value of circa 4, indicating that as the water content within the solvent increases, the probable absorption of water to the nanopipette walls dominates the ion transport properties therein.

**Figure 6:**
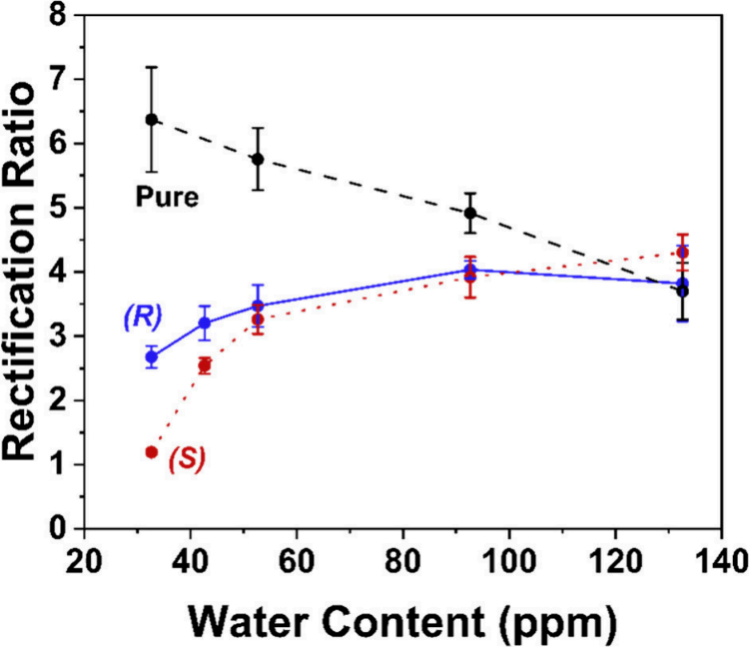
Rectification ratio as a function of water content (measured by Karl-Fisher titration) in (black) pure electrolyte and electrolyte containing either (blue) (*R*)- or (red) (*S*)-4-methoxy-α-methylbenzylamine. *I–V* responses are measured in 0.5 mM TEATFB in MeCN, using bare quartz nanopipettes with radii of ∼50 nm. 0.5 mM enantiomer is added to the external bulk electrolyte bath for detection. Error bars indicate the standard error from a measurement of six or more unique nanopipettes.

### Determination of Enantiomeric Excess Following Asymmetric Catalysis

With the aim of further exploiting the practical applications of our technology for enantiomeric discrimination, we hypothesized that ee values where (*S*)- is the major enantiomer could be accessed by changing the electrolyte composition from an organic to an alkali metal cation, to alter the initial ordering of MeCN molecules along the inner nanopore surface prior to enantiomer exposure and adsorption. In a recent study by Souna *et al*.,^60^ and earlier studies by Polster *et al*.^61^ and Berne *et al.*,^62^ MeCN molecules were shown to form lipid-like bilayers on silica surfaces, meaning in neat MeCN, the surface exhibits an effective negative charge. Polster *et al*.^61^ described that at high enough concentrations of lithium perchlorate (LiClO_4_) the effective surface charge becomes positive, due to the interaction of the electrolyte ions with the bilayer. This effect is dependent on the electrolyte species, and in NaClO_4_, due to the larger size of Na^+^, an effective positive surface potential occurs at a much higher electrolyte concentration.^61^ Thus, we postulated that measurements in NaTFB, vs. TEATFB would significantly affect the effective surface potential of the nanopore, changing the initial state of the sensor. Nanopores measured in neat NaTFB are much less rectifying (RR of 2.7 ± 0.3) than in TEATFB (RR of 5.4 ± 0.3) supporting this hypothesis (Figure 7a). Consequently, exposure to (*R*)-4-methoxy-α-methylbenzylamine results in suppression of ICR to 0.4 ± 0.03. Whereas in the presence of the (*S*)-enantiomer, the RR is higher, at 1.7 ± 0.2. This is a reversal of the response observed in TEATFB, where (*S*)- suppresses the signal and (*R*)- causes a lesser change (Figure 1a). Figure 7b shows a similar, but opposite, trend to TEATFB (Figure 2) as a function of ee [% (*S*) – % (*R*)]. Negative rectification increases exponentially towards a maximum at 80%, after which a gradual reversal in the direction of ICR towards 0.4 occurs as the enantiomeric excess of the (*S*)-enantiomer decreases.

**Figure 7:**
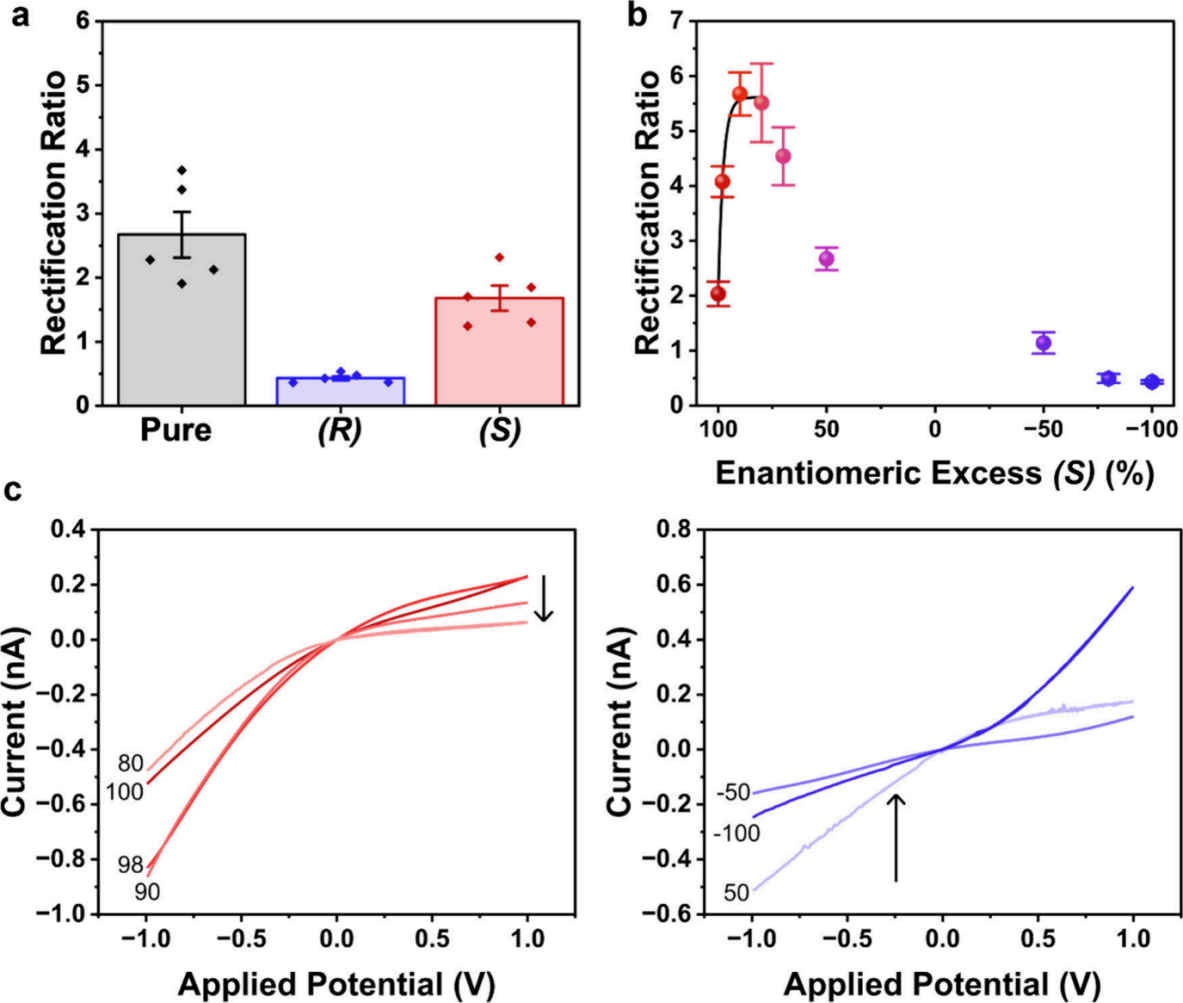
(a) Rectification ratio measured in (black) pure NaTFB electrolyte and electrolyte containing either (blue) (*R*)- or (red) (*S*)-4-methoxy-α-methylbenzylamine. (b) Rectification ratio as a function of enantiomeric excess of (*S*)-4-methoxy-α-methylbenzylamine [% (*S*) – % (*R*)] with (c) representative *I–V* responses at different enantiomeric excess values. *I–V* responses are measured in 0.5 mM NaTFB in MeCN, using bare 50 nm radius quartz nanopipettes. 0.5 mM enantiomer is added to the external bulk electrolyte bath for detection. Samples of different enantiomeric excess are achieved by mixing stock solutions of commercially available (*R*)- and (*S*)-4-methoxy-α-methylbenzylamine in different ratios, with dilution to an overall analyte concentration of 0.5 mM. Representative error bars indicate the standard error from a measurement of five or more unique nanopipettes.

Unfortunately, enantiomeric discrimination of duloxetine hydrochloride for cases when the (*S*)-enantiomer is in significant excess was not possible using this method. Upon addition of duloxetine hydrochloride to the electrolyte solution, a fine dispersion was observed, which was difficult to remove despite multiple filtrations through a 0.2 μm syringe filter. We believe NaCl is formed through an ion exchange between NaTFB and HCl, affecting the composition of the electrolyte solution, and thus, the effectiveness of the sensor. Under these conditions, the nanopores were frequently blocked, and/or gave inconsistent RR values with no observable trends as a function of ee. In future work we will seek to identify an electrolyte composition compatible with hydrochloride salts of drug compounds for this purpose.

Given the promising results of enantiopurity determination using commercial samples, we sought to explore the applicability of the sensor for quality control following a synthesis carried out both asymmetrically and racemically (Scheme 1). The reaction studied was a decarboxylative asymmetric allylic alkylation (DAAA) of an α-allyl-α-aryl lactone employing tris­(dibenzylideneacetone)­dipalladium (Pd_2_(dba)_3_) in the presence of the chiral (*R*,*R*)-ANDE*N*-phenyl Trost ligand or achiral 1,2-bis­(diphenylphosphino)­ethane (DPPE) (Scheme 1).^63^ Previously, we have extensively investigated this process as a methodology to prepare sterically hindered, all-carbon quaternary stereocenters possessing α-allyl-α-aryl motifs.^64–67^ This reaction is highly enantioselective for the formation of the (*S*)-product, as previously confirmed using X-ray crystallography.^63^

**Scheme 1:**
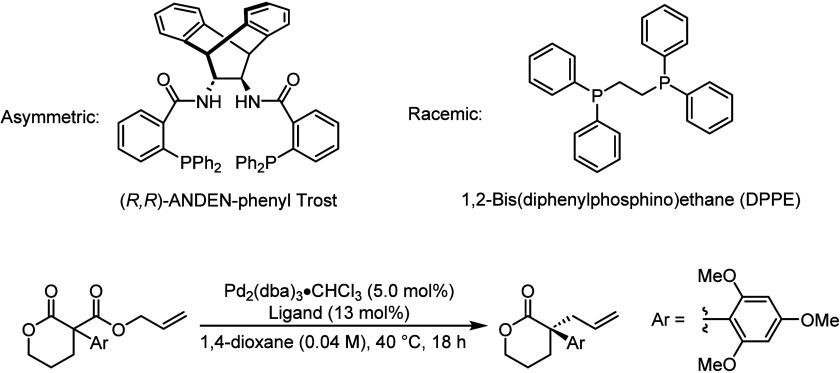
Synthesis of an α-Allyl-α-Aryl Lactone *via* Decarboxylative Asymmetric Allylic Alkylation Employing Pd_2_(dba)_3_ and (*R*,*R*)-ANDEN-Phenyl Trost Ligand (Asymmetric) or 1,2-Bis­(diphenylphosphino)­ethane (Racemic)

The enantiomeric excess of each product was determined using supercritical fluid chromatography, as shown in Figure S5, and the nonlinear *I–V* response of each was compared. The asymmetric or racemic products (0.5 mM) were dissolved in both TEATFB and NaTFB in MeCN (0.5 mM), and ICR was measured using bare quartz nanopipettes as previously described.

The asymmetric transformation presented in Scheme 1 is highly enantioselective for the formation of the (*S*)-enantiomer. We therefore anticipated that using an NaTFB supporting electrolyte would be required to achieve discrimination between the racemic and asymmetric product, due to the major suppressive effect of the (*S*)-enantiomer in TEATFB observed with the model compounds (Figure 1). This was found to be true, and a far greater degree of separation in RR between the racemic and asymmetric product was observed in NaTFB compared to TEATFB (Figure 8a,b). Subsequent enantiomeric excess determination was performed by mixing the product of the asymmetric and racemic synthetic processes in different ratios in NaTFB. Figure 8c shows a decrease in RR as ee increases, and importantly, discrimination in the 90% to 100% ee region.

**Figure 8:**
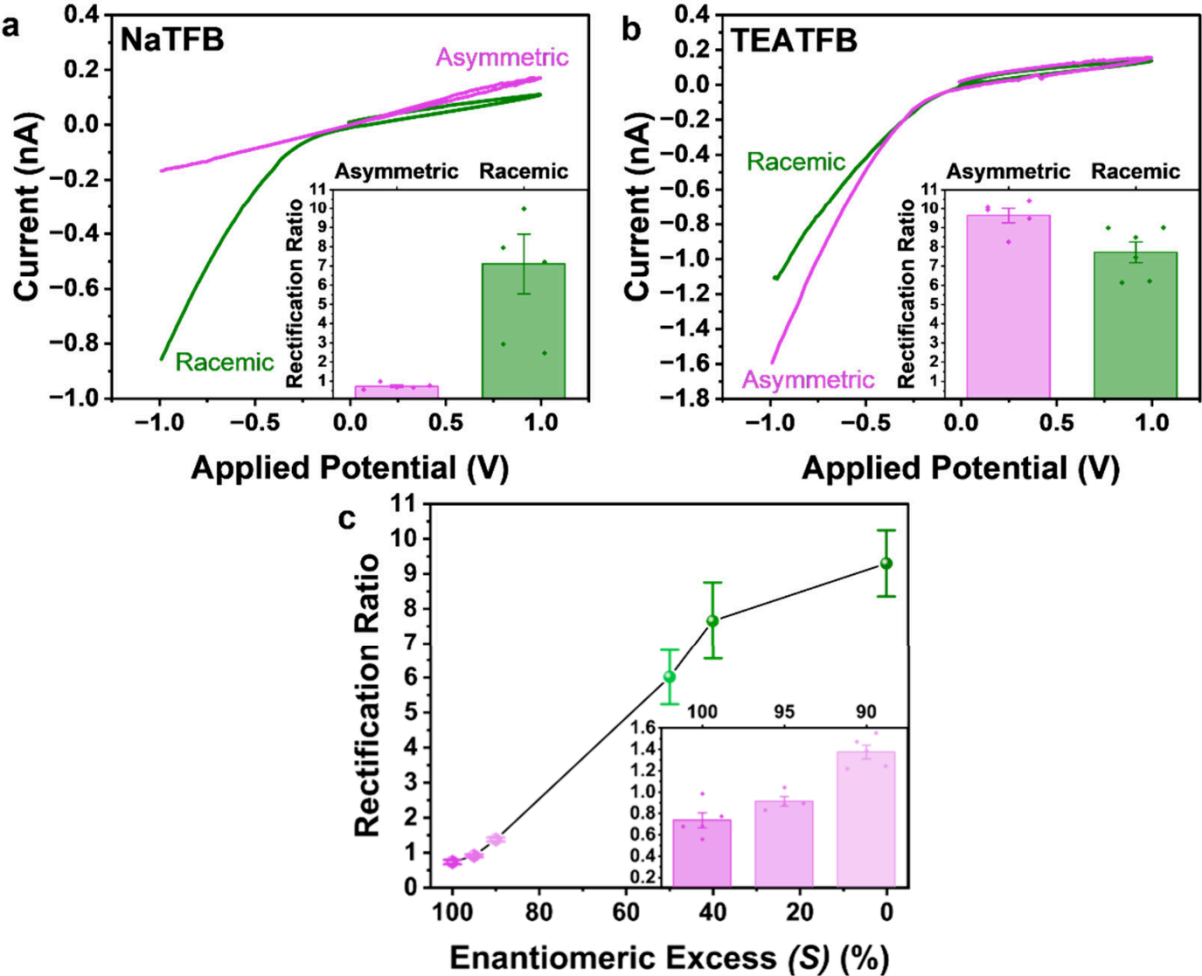
Representative *I–V* responses and rectification ratio (inset) measured in electrolyte containing (pink) asymmetric and (green) racemic synthetic products in (a) NaTFB and (b) TEATFB. (c) Rectification ratio as a function of enantiomeric excess of the real sample [% (*S*) – % (*R*)], with the inset showing the 90–100% ee region. Solutions of different enantiomeric excess are achieved by mixing the racemic and asymmetric samples in different ratios, followed by dilution to 0.5 mM analyte concentration. ee values of the racemic and asymmetric samples are confirmed by supercritical fluid chromatography (Figure S5). *I–V* responses are measured in 0.5 mM electrolyte in MeCN, using bare 50 nm quartz nanopipettes. 0.5 mM of sample is added to the external bulk electrolyte bath for detection. Representative error bars indicate the standard error from a measurement of five or more unique nanopipettes.

### Current Limitations of the Sensing Methodology

We note here that the enantiomeric pairs of each compound described, while are all distinguishable using our technology, do produce different ICR responses. It would therefore be necessary to build specific calibration curves for a compound’s pair of enantiomers (or with an enantiopure and racemic sample) prior to determining enantiomeric excess. The different responses for each compound likely arise from their different chemical properties and functional groups. These will impact both their adsorption properties at the quartz interface, as well as their solvation within the acetonitrile electrolyte. For example, the aryl functional group on the synthesized product shown in Scheme 1 contains three electron-donating methoxy groups, which greatly increases electron density in the aryl ring. This will certainly have a significant effect on the molecule’s interactions with the hydroxy-terminated quartz surface, and with proximal solvent molecules. It is therefore noted that the nanopipette response to enantiomers is highly dependent on their chemical structure, and future work is required to determine if this enantiomer discrimination technique is widely applicable over a large library of different compounds with varying electron-donating and electron-withdrawing groups. In addition, the observed nonlinearity in calibration curves means that in an unknown solution, an identical response would be observed at an ee of ∼60% and ∼95% (Figures 2 and 3). This is not unusual in MeCN due to the nonlinear surface charge, and was also observed in our previous calibration curves for the detection of trace metals using probe-functionalized nanopores.^68^ For this reason, in its present form, the sensor described here is only effective in the 90–100% ee range which fortuitously is the range of relevance for quality control of high purity asymmetric synthesis.^69^

Achieving a wider practical working range will require exploration of a wide range of alternative experimental conditions, such as supporting electrolyte, solvent and pore size. This is an important avenue for future work, as these parameters are known to have significant and interesting effects on ICR.^70^ Mechanistic insight may be achieved by studying aprotic solvents of different polarity, and as a result different effective surface charges and degrees of ordering, such as dichloromethane (DCM) which exhibits a lower effective surface charge than MeCN.^38^ Siwy and coworkers have demonstrated the importance of solvent chirality and electrolyte concentration in propylene carbonate nanopore systems,^41,42^ and exploiting the high degree of ordering in racemic propylene carbonate may result in improved enantiomer discrimination using our system. In MeCN, we have shown amplification of ICR in the mid to low electrolyte concentration range (circa 1 mM),^38^ and further studies of the enantiodependent nanopore response at different electrolyte concentrations pre- and post-maximum may also allow for further optimization of our system. Finally, it should also be noted that because ICR measurements in nanopipettes are also highly sensitive, this does also imply higher sensitivity to impurities, which could dominate the response depending on their structure and charge. The work here used high purity solvents and dedicated glassware to prevent exposure to laboratory contaminants. In future work, we plan to spike pure enantiomers with known impurities, to determine their impact on the response.

## Conclusion

In conclusion, we have developed a low-cost technology that can discriminate between enantiomers within minutes, with minimal sample volume, using only bare quartz nanopipettes and simple electronics capable of recording a current-voltage response. The determination of enantiomeric excess from mixtures of enantiomers has also been demonstrated and is effective up to ee values of 99%. We speculate a detection mechanism based on enantiomerically selective adsorption of molecules to the internal quartz nanopore walls, noting that previous works have shown preferential adsorption onto silica powders from non-aqueous solvent. The adsorption of chiral solutes results in a change in solvent ordering, giving rise to a change in nanopore surface charge, thus a change in observed rectification ratio. We believe this work may have the potential to inspire a new class of miniaturized, low-cost chemical characterization techniques for pharmaceutical quality control and enantiopurity determination with minimal requirements for reagents, sample size and facilities.

## Supplementary Material



## Notes

Full experimental procedures and characterization data; further experimental data (PDF)
