# Supplementary material for: Determination of Enantiomeric Excess in Confined Aprotic Solvent
Source: ACS Electrochem. 2025 Feb 26;1(6):928–39. doi: 10.1021/acselectrochem.4c00219 (PMC12147146; doi:10.1021/acselectrochem.4c00219)
Supplement: Supplementary file 1 [file ec4c00219_si_001.pdf]

SUPPORTING INFORMATION:

Determination of Enantiomeric Excess in Confined Aprotic Solvent

Emer B. Farrell, Fionn McNeill, Dominik Duleba, Adria Martínez-Aviño, Patrick J. Guiry, Robert P. Johnson*

School of Chemistry, University College Dublin, Belfield, Dublin D04 N2E5, Ireland
*robert.johnson@ucd.ie

Contents

Characterization of Nanopipettes 2
Additional Ion Current Rectification Data 3
Details of Asymmetric and Racemic Synthesis 5
Supporting References 7

Characterization of Nanopipettes

Nanopipette radii were determined by recording current-voltage traces using 0.1 M KCl electrolyte in deionised water. Nanopipettes were backfilled with electrolyte, and a Ag/AgCl wire working electrode was inserted. The nanopipettes were placed in a bulk electrolyte bath containing a Ag/AgCl wire reference electrode, such that the tip was submerged, and current-voltage traces were measured. The applied potential was swept from -1 to 1 V with respect to the reference electrode, at a scan rate of 0.1 V s^{-1}. A linear fit was applied to the resulting CV using EC-Lab software, and the slope was used to calculate the nanopipette radius based on equation S1.[2, 3]

$$r = \frac{1}{\kappa R}\left(\frac{1}{\pi \tan\frac{\theta}{2}} + \frac{1}{4}\right) \tag{S1}$$

Where **k** is the electrolyte conductivity, **θ** is the cone angle and ***R*** is the nanopipette resistance. By inserting resistance, the inverse of conductivity (obtained from the slope of the CV), the nanopipette radius (r) can be determined, assuming a constant cone angle between nanopipettes, and excluding the effect of nanopipette wall surface charge.

Pore dimensions and geometry were confirmed by imaging a subset of pores with scanning electron microscopy (SEM).

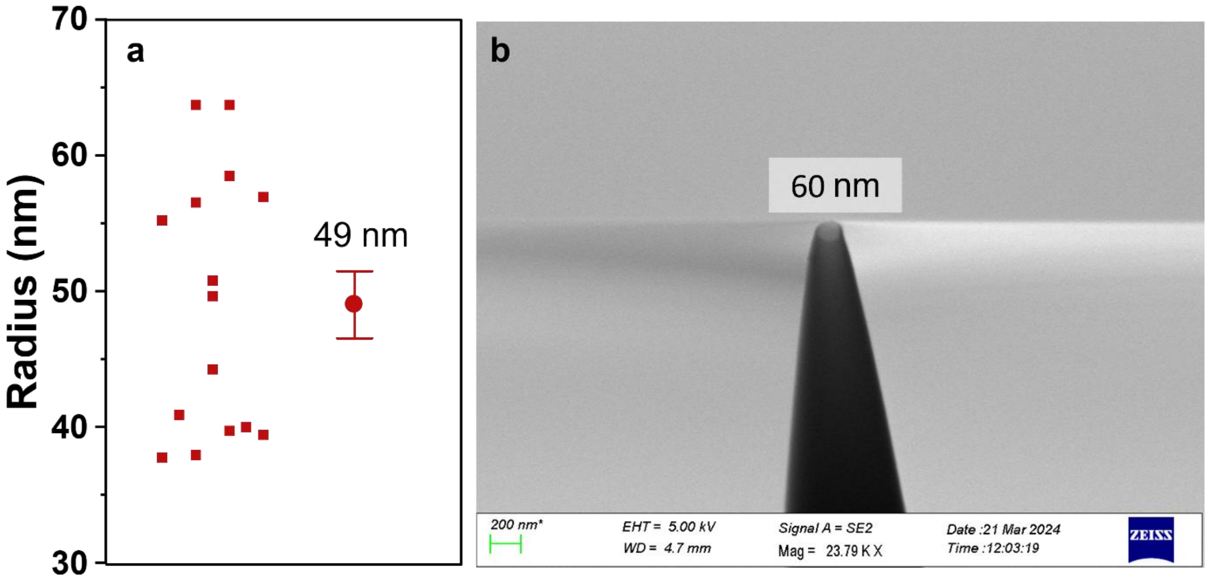

Figure S1: The **(a)** electrochemical and **(b)** SEM characterization of nanopipette tip radius size in nm.

Additional Ion Current Rectification Data

Stability and Reproducibility of the Current-Voltage Response

Additional experimental data demonstrating the reproducibility and stability of nanopipette measurements in the absence and presence and enantiomer analyte are presented in Figures S3 and S4 below.

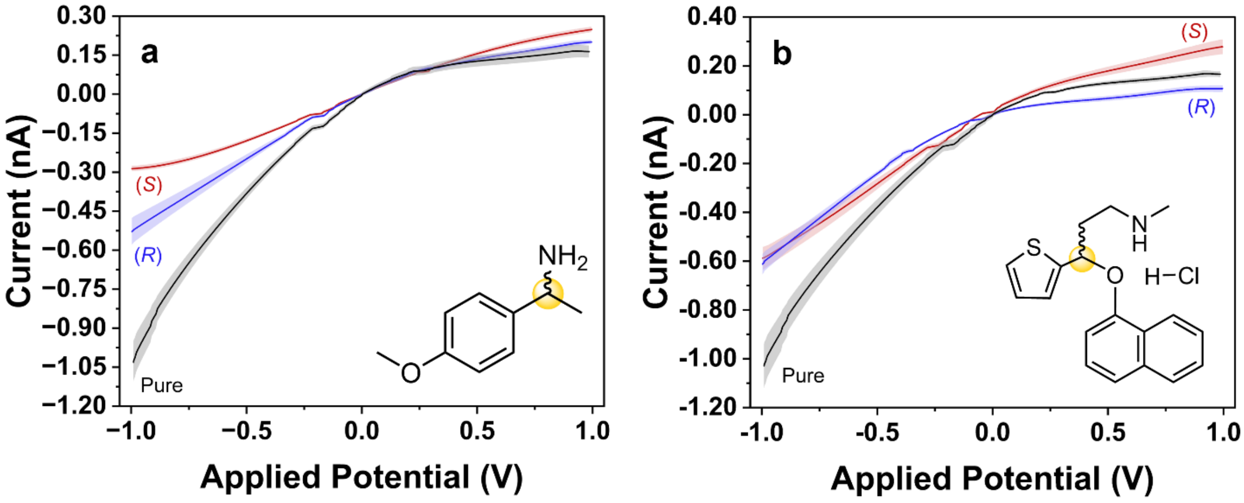

Figure S2. Average CV responses across six unique devices measured in (black) pure electrolyte and electrolyte containing (blue) (*R*)-, and (red) (*S*)-enantiomer for (**a**) 4-methoxy-α-methylbenzylamine and (**b**) duloxetine hydrochloride. All CVs are measured in 0.5 mM TEATFB in MeCN, using bare radius 50 nm quartz nanopipettes. 0.5 mM of enantiomer is added to the external bulk electrolyte bath for detection. The shaded region is the standard error at each data point for six individual nanopipettes.

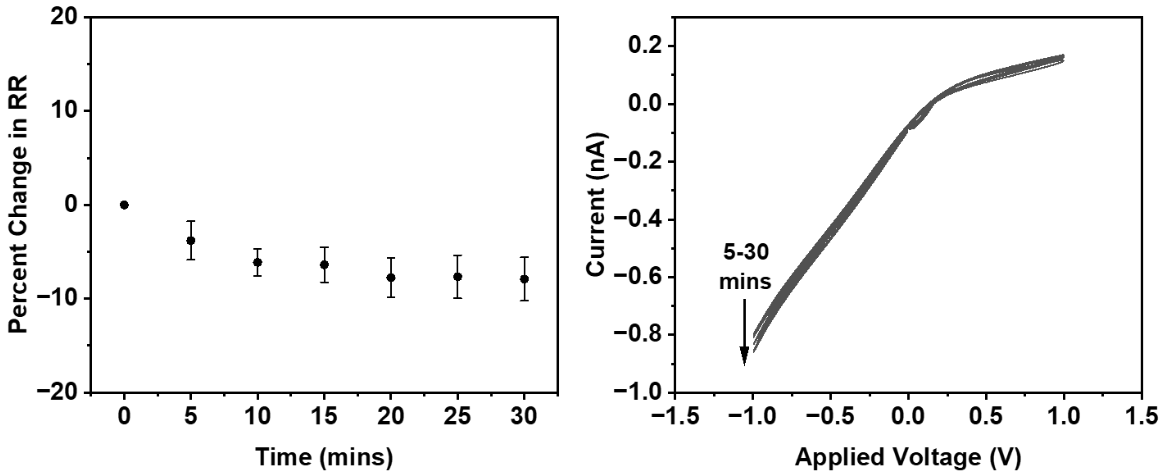

Figure S3. The percent change in rectification ratio as a function of time measured and representative CV response as function of time for 0.25 mM (*S*)-4-methoxy-α-methylbenzylamine. All CVs are measured in 0.5 mM TEATFB in MeCN, using bare radius 50 nm quartz nanopipettes. Error bars indicate the standard error from a measurement of three unique nanopipettes.

Testing for Enantiomer Adsorption

To elucidate the mechanism of enantioselectivity with quartz nanopipettes, we conducted experiments in which post exposure to enantiomer analyte, the nanopipettes were flushed with pure electrolyte and then re-measured. Our data indicated that the suppressed ICR response remained even after removal of the analyte from the solution phase, which implies strong adsorption to the quartz nanopipette walls.

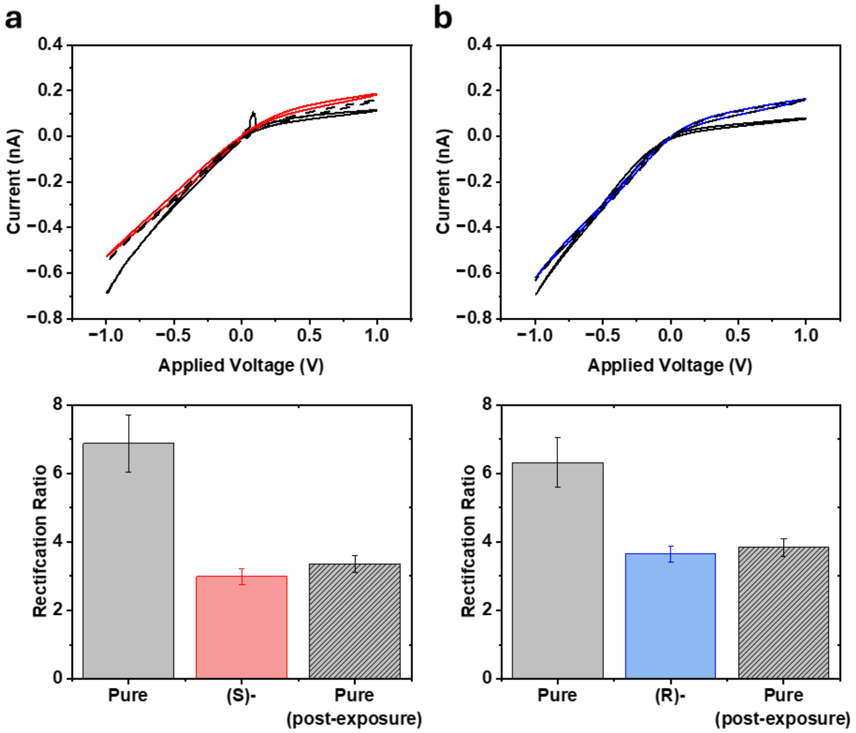

Figure S4. Representative CV response and rectification ratios measured in (black) pure electrolyte, and electrolyte containing either 0.25mM (**a**, red) (*R*)-, or (**b**, blue) (*S*)-4-methoxy-α-methylbenzylamine, followed by rinsing of the nanopipette and re-measurement (dashed-black) in pure electrolyte. All CVs are measured in 0.5 mM TEATFB in MeCN, using bare radius 50 nm quartz nanopipettes. Error bars indicate the standard error from a measurement of six unique nanopipettes.

Details of Asymmetric and Racemic Synthesis

Synthesis of 3-Allyl-3-(2,4,6-trimethoxyphenyl)tetrahydro-2*H*-pyran-2-one *via* Decarboxylative Asymmetric Allylic Alkylation Employing $Pd_2(dba)_3$ and (R,R)-ANDEN-phenyl Trost ligand (asymmetric) or 1,2-Bis(diphenylphosphino)ethane (racemic).

Asymmetric: In a 10 mL, flame-dried Schlenk tube, $Pd_2(dba)_3{\cdot}CHCl_3$ (5.0 mol %) (5.2 mg), (*R,R*)-ANDEN phenyl Trost ligand (13.0 mol %) (10.6 mg) and α-aryl-β-oxo allyl ester (1 equiv.) (35 mg) were dissolved in 1,4-dioxane (0.04 M) (2.5 mL). The reaction mixture was stirred under a N_2 atmosphere at 40 °C for 18 h. The reaction solvent was filtered through a celite plug washing through with 3 mL of DCM. The solvent was removed in vacuo and the crude residue was purified by flash column chromatography (10 % EtOAc in Cyclohexane increasing to 25%). The product was collected as an off-white oil.

Racemic: In a 25 mL, flame-dried Schlenk tube, $Pd_2(dba)_3{\cdot}CHCl_3$ (5.0 mol %) (10.4 mg), DPPE ligand (13.0 mol %) (10.4 mg) and α-aryl-β-oxo allyl ester (1 equiv.) (70 mg) were dissolved in 1,4-dioxane (0.04 M) (5 mL). The reaction mixture was stirred under a N_2 atmosphere at 40 °C for 18 h. The reaction solvent was filtered through a celite plug washing through with 3 mL of DCM. The solvent was removed in vacuo and the crude residue was purified by flash column chromatography (10% EtOAc in Cyclohexane increasing to 25%). The product was collected as an off-white oil.

Analysis: The product in each case was analyzed by ^{1}H-NMR and matched literature data.[4]
Enantioselectivity was determined by using supercritical fluid chromatography: (Chiralcel IJ-3, $scCO_2$/MeOH, 99/1 to 95/5 gradient over 10 min, 3mL/min): R_t: 2.99 (major) and 3.19 min (minor).

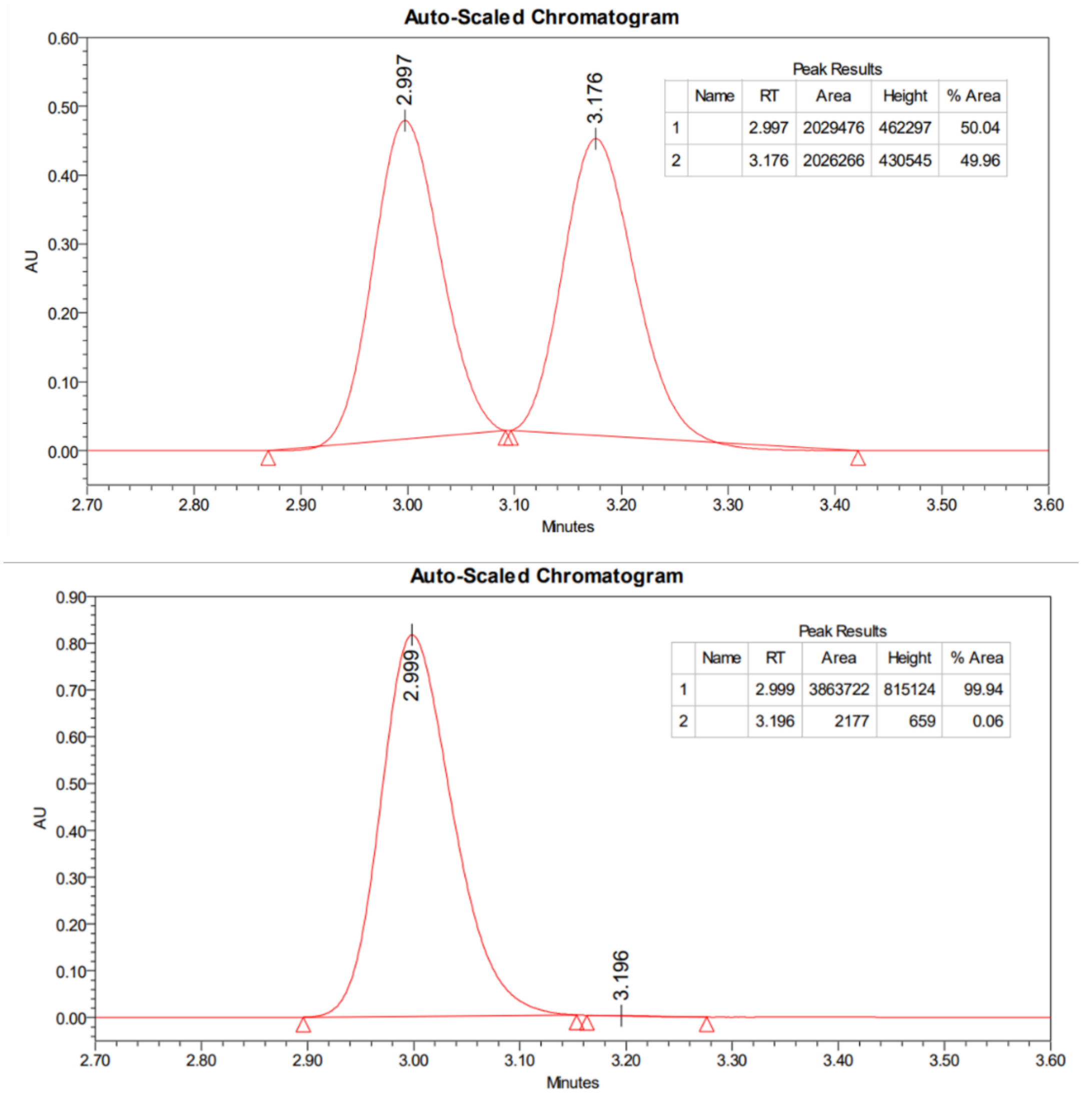

Figure S5. Supercritical fluid chromatograms for asymmetric and racemic synthetic products.

Supporting References

(1) Zalesskiy, S. S.; Ananikov, V. P. $Pd_2(dba)_3$ as a Precursor of Soluble Metal Complexes and Nanoparticles: Determination of Palladium Active Species for Catalysis and Synthesis. *Organometallics* **2012**, *31* (6), 2302-2309. DOI: 10.1021/om201217r.
(2) Del Linz, S.; Willman, E.; Caldwell, M.; Klenerman, D.; Fernandez, A.; Moss, G. Contact-Free Scanning and Imaging with the Scanning Ion Conductance Microscope. *Anal. Chem.* **2014**, *86* (5), 2353-2360. DOI: 10.1021/ac402748j.
(3) Perry, D.; Momotenko, D.; Lazenby, R. A.; Kang, M.; Unwin, P. R. Characterization of Nanopipettes. *Anal. Chem.* **2016**, *88* (10), 5523-5530. DOI: 10.1021/acs.analchem.6b01095.
(4) James, J.; Guiry, P. J. Highly Enantioselective Construction of Sterically Hindered α-Allyl-α-Aryl Lactones via Palladium-Catalyzed Decarboxylative Asymmetric Allylic Alkylation. *Acs. Catal.* **2017**, *7* (2), 1397-1402. DOI: 10.1021/acscatal.6b03355.
